# Magnetoreception—A sense without a receptor

**DOI:** 10.1371/journal.pbio.2003234

**Published:** 2017-10-23

**Authors:** Gregory C. Nordmann, Tobias Hochstoeger, David A. Keays

**Affiliations:** Research Institute of Molecular Pathology, Vienna, Austria

## Abstract

Evolution has equipped life on our planet with an array of extraordinary senses, but perhaps the least understood is magnetoreception. Despite compelling behavioral evidence that this sense exists, the cells, molecules, and mechanisms that mediate sensory transduction remain unknown. So how could animals detect magnetic fields? We introduce and discuss 3 concepts that attempt to address this question: (1) a mechanically sensitive magnetite-based magnetoreceptor, (2) a light-sensitive chemical-based mechanism, and (3) electromagnetic induction within accessory structures. In discussing the merits and issues with each of these ideas, we draw on existing precepts in sensory biology. We argue that solving this scientific mystery will require the development of new genetic tools in magnetosensitive species, coupled with an interdisciplinary approach that bridges physics, behavior, anatomy, physiology, molecular biology, and genetics.

## Introduction

**Fig 1:**
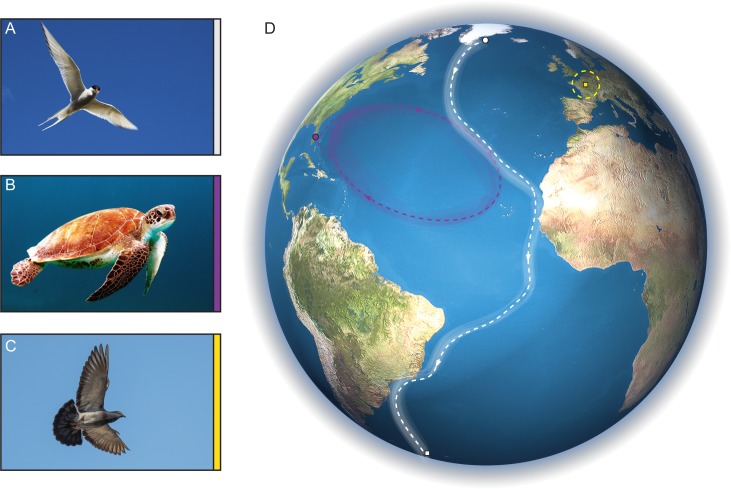
Animals that undertake astonishing journeys. Image of an (A) arctic tern (*Sterna paradisaea)*, (B) a loggerhead turtle *(Caretta caretta*), and (C) a rock pigeon (*Columba livia*). (D) Representation of the Earth that shows the journeys undertaken by these 3 species. White tracks show the migratory route of the arctic tern. Greenlandic colonies leave their breeding grounds (white circle) in August and follow migratory routes along the Brazilian coast to spend the austral Winter (December to April) in regions of the Antarctica (white square). Purple tracks show the migratory route of loggerhead turtles) that hatch off the east coast of Florida (purple line), circle the North Atlantic gyre, before returning to the very same stretch of coastline for nesting. Yellow track shows the homing range of a pigeon (approximately 500 km), around the city of Paris, France.

Invented by the Chinese between 200 BC and 100 AD, the compass was first exploited by mariners as a tool around 1000 AD [1]. Coupled with early star charts, it ushered in an era of exploration as humanity set sail for the horizon [2]. Yet for thousands, perhaps millions of years prior, evolution had equipped life on the planet with a biological global positioning system that was far superior to those early navigational aids. A system that guided the artic tern on its annual migration from its rookeries in Greenland to the plentiful feeding grounds in Weddal Bay in Antarctica (and back again; Fig 1A and 1D) [3]. A biological apparatus that enabled female loggerhead turtles after spending their juvenile life at sea, to return to the very same beach where they hatched more than 10 years ago (Fig 1B and 1D) [3]. A sense that was utilized by pigeons as they delivered microfilm, strapped precariously to their tiny feet, as the city of Paris lay siege to the Prussians in the 19th century (Fig 1C and 1D). It was the Russian zoologist Alexander von Middendorff who was amongst the first to speculate that these animals might use the Earth's magnetic field as a navigatory cue:

…like a magnetic needle for ships, those sailors of the air possess an inner magnetic feeling, which might be linked to the galvanic-magnetic flows… (Middendorff 1855)

Yet, it would take more than a century for this scientific intuition to become fact. It was not until the 1960s, with the maturation of behavioral biology from a descriptive to an experimental discipline, that Friedrich Merkel and Wolfgang Wiltschko were able to conclusively show magnetic field-dependent behavior in a migratory bird, the European robin (*Erithacus rubecula*). By using small circular arenas they modified the preferred direction of orientation during migratory restlessness by the application of artificial magnetic fields [4]. Their initial demonstration of magnetosensory behavior in birds was based on crucial experimental features that had previously been absent: a reductionist approach, quantitative parameters, and controlled environmental conditions. With the evolution of these behavioral tests and the expansion of the field in the last 20 years, the number of species identified with a magnetic sense has grown dramatically. The list now includes fish, turtles, mammals, birds, insects, and even bacteria [5–10]. Although the existence of the sense can no longer be disputed, we understand little of the cells, molecules, and mechanisms that underlie it. Before we discuss how animals detect magnetic fields, we must first appreciate what they are detecting (Box 1).

**Box 1. What is the stimulus?**

The Earth’s magnetic field is believed to arise from the motion of its conducting fluid core, which is rich in iron [11]. It is a vector quantity that can be considered to have 3 components: (1) an inclination; (2) a declination; and (3) an intensity. Magnetic field lines emerge from the planet forming an angle in relation to the Earth's surface that varies with the latitude. This is referred to as the inclination of the field. For instance, the vector points vertically towards the sky at the south pole (−90°), runs parallel to the surface at the magnetic equator (0°), and enters the Earth at 64° 19ʹ in Paris. In contrast, the declination refers to the angle of the magnetic field lines with respect to true geographic North, reflecting the direction a compass needle points. The intensity of the field (25-65nT), represents the density of magnetic field lines and is measured in Gauss or Tesla. It is influenced by the distribution of ferromagnetic materials in the Earth's crust, and therefore can be shown as a topographic map of magnetic intensities. As the inclination, declination, and intensity of the geomagnetic field are distinct geophysical parameters, each could be used as a navigation aid by migratory animals.

### How might animals detect the magnetic field?

Analogous to vision, touch, or hearing, it is predicted that the magnetic sense relies on a specialized population of cells that are associated directly, or indirectly, with the central nervous system [12] We expect that these cells express a suite of molecules that are necessary for the transduction of a magnetic stimulus into a cellular response. We anticipate that this involves the modulation of intracellular signaling cascades, which in turn mediates neurotransmitter release, the activation of secondary neurons, and the subsequent integration of information within specific circuits of the central nervous system [13]. But, how exactly would the transduction process work? Currently, there are 3 dominant hypotheses that are being tested. The first predicts that magnetic fields are detected by a mechanically sensitive magnetite-based magnetoreceptor. The second hypothesis relies on a light-sensitive, chemical-based mechanism, and the third idea is dependent on an anatomical structure that would enable electromagnetic induction. It should be emphasized that these concepts are not mutually exclusive, and that animals may have evolved multiple mechanisms to detect different components of the field.

### A mechanically sensitive magnetite-based magnetoreceptor

**Fig 2:**
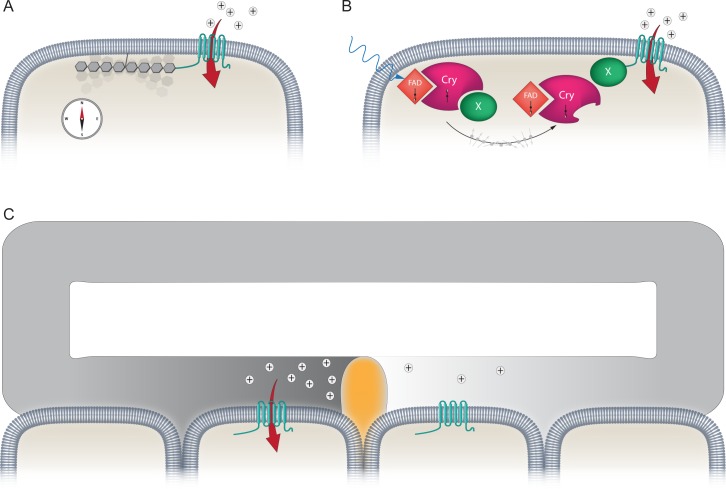
Three mechanisms proposed to underlie the magnetic sense. (A) Image depicting a mechanically sensitive magnetite-based magnetoreceptor. Magnetite crystals (shown here as a chain) are attached to the plasma membrane via a cytoskeletal linker. This linear arrangement of single-domain crystals attempts to align with the Earth's geomagnetic field (like a compass needle), and thereby exerts a torque force on a mechanosensitive channel (shown in teal). This transiently activates the channel leading to cation influx (red arrow) and membrane depolarization. (B) Diagram showing a light-sensitive chemical-based magnetoreceptor. Blue light (shown with a blue arrow) induces the formation of long-lived radical pairs between Cry and the cofactor FAD. The spin state of these electrons interconverts between an antiparallel (↑↓) or parallel (↓↓) state, depending on the local magnetic environment. This, in turn, influences the biochemical or structural properties of Cry, resulting in the activation of an unknown signaling molecule (X) that modulates ion channel permeability. (C) Magnetoreception based on electromagnetic induction. This hypothesis relies on an accessory structure that transforms magnetic stimuli into an electrical information. Depicted is one semicircular canal of a vertebrate, filled with cation-rich endolymph, and sensory cells located on either side of the cupula (shown in orange). If the animal moves so that rotation occurs around an axis in the plane of a semicircular canal, there will be no displacement of the endolymph but electromagnetic induction could occur. Depending on the intensity and orientation of the external magnetic field, this will induce an electromotive force in the conductive endolymph. This results in the separation of charges within the circuit, inducing cation influx through highly sensitive voltage-gated ion channels (shown in teal). Cry, cryptochrome; FAD, flavin adenine dinucleotide.

In many sensory systems, the receptor proteins involved in transducing the signal are directly influenced by the incoming stimulus and undergo structural rearrangements upon activation. For instance, it is known that the mechanosensitive channel Piezo1 directly senses forces at the plasma membrane, resulting in cation influx in response to pressure [14,15]. Might magnetosensation rely on a similar mechanism? There are 2 issues that arise here. First, the Earth's magnetic field is weak, and secondly, proteins exhibit very low magnetic susceptibility because they are primarily composed of carbon, nitrogen, and oxygen. If a membrane protein is to undergo a conformation change in an Earth strength field, it would need to be coupled to a ferrimagnetic structure made of an iron oxide such as magnetite (Fe_3_O_4_) [16]. This conceptually simple idea is tenable because it is known that a number of species are able to form biogenic magnetite. The best example are magnetotactic bacteria, which generate a chain of intracellular magnetite crystals [5,17]. They employ this internal compass needle to guide their swimming along the incline of the magnetic field vector to deeper waters with favorable redox conditions. Such observations have led to the formulation of the magnetite-based hypothesis for magnetoreception, which predicts the existence of a mechanosensitive channel attached to magnetite crystals (Fig 2A) [18,19]. This has motivated many researches to search for magnetite in vertebrates, employing methods such as superconducting quantum interference device SQUID magnetometry, energy filtered transmission electron microscopy (EFTEM), magnetic purification techniques, and the histological stain Prussian Blue (which labels ferric iron [Fe^3+^]). To date, this search has not been marked by success. Macrophages have been mistaken for magnetoreceptors [20,21], iron contamination from the laboratory environment has led investigators astray [22–24], and the ability to locate nanometer size crystals in a whole organism has proved an extremely challenging task [25]. An alternative approach has been to employ bioinformatic methods to search for eukaryotic orthologues of the well characterized Mam proteins that are required for magnetite synthesis in magnetotactic bacteria. Although some domain structures are conserved (e.g., MamP has a PDZ domain), the available sequence data suggest there are no genuine homologues of the Mam proteins in eukaryotic magnetosensitive species [17,26,27]. One thing is clear, if indeed a magnetite-based magnetoreceptor exists, it's not going to be easy to find.

### A light-sensitive chemical-based compass

Alternatively, it is conceivable that the geomagnetic field could influence biochemical reactions within a receptor cell analogous to detection of light by visual pigments. Rhodopsin, the best characterized photoreceptive protein, is reliant on a chromophore (11-*cis*-retinal), which undergoes a structural change on the absorption of a photon. This alters the biochemical properties of the opsin, resulting in an activation of G-proteins, and alteration of downstream signaling cascades [28]. Behavioral data in European robins have demonstrated that magnetic orientation requires light in the blue/green spectrum, supporting a light-dependent hypothesis for magnetoreception [29]. This concept predicts that the geomagnetic field can influence the spin state of light-induced radical pairs and thereby modulate the outcome of biochemical reactions [30–32]. This is possible because electrons possess a quantal property known as spin angular momentum, and consequently radical pairs can be ordered in a parallel (↓↓) or antiparallel state (↑↓). It is known that external magnetic fields can influence the interconversion between these different spin states, which in turn could influence the reactivity of a particular molecule [33,34]. Consistent with the hypothesis, a number of behavioral studies in vertebrates have shown that low-intensity broadband electromagnetic fields (which influence electron spins) disrupt magnetic orientation [35]. It has been proposed that the cryptochromes (Crys), which have a conserved photolyase domain that binds the flavin adenine dinucletotide (FAD) cofactor, possess the molecular attributes to function as a magnetosensor. According to the favored model, a long-lived radical pair is formed when light induces the transfer of electrons to this FAD moiety along a triad of tryptophan residues in the Cry molecule (Fig 2B) [31,36].

Genetic studies in invertebrates have supported the role of Cry in magnetically sensitive behavior. For instance, Fedele and colleagues have shown that low frequency electromagnetic fields (300 μT, 50 Hz) shortened the circadian period and induced hyperactive behavior in *Drosophila*, which is abolished in the absence of Cry [37]. Although these results indicate that Cry is required for magnetic fields to influence certain behaviors in flies, it is questionable whether these animals possess a true magnetic sense analogous to migratory birds. Furthermore, given that Crys are expressed in all major organs in birds and are known to play a role in regulating circadian phenotypes in vertebrates, it is currently unclear how specificity would arise with respect to magnetic information and how it would be distinguished from a circadian input [38,39]. Moreover, we still don't know which cells are associated with a light-dependent chemical compass. Although it is widely assumed that the primary sensors reside in the retina, no report has convincingly shown that a particular cell type (e.g., cones) is sensitive to magnetic stimuli. While we have good reason to believe a radical-pair based magnetic sense exists, it is apparent that we still understand little with respect to the receptive cells, the molecules, and the downstream signaling cascade.

### An anatomical structure that would enable electromagnetic induction

Finally, the detection of magnetic fields might rely on accessory structures that convert the magnetic field into another stimulus. For instance, terrestrial vertebrates do not detect gravity directly, but rather rely on a bed of calcium carbonate otoliths which apply a force on a population of sensory hair cells [40]Similarly, the auditory system exploits a complex array of accessory structures that convert airborne sound pressure waves into gating spring tension on mechanosensitive ion channels in hair cells [41] As magnetism and electricity are inseparable, it is conceivable that a secondary structure might convert information about the Earth's magnetic field into an electric stimulus. This proposition, which reflects Faraday’s law of electromagnetic induction, predicts that movement of an animal in a fixed magnetic field would induce an electromotive force in a conductor [42]. If a circuit exists, a change in electric potential could be detected by cells expressing voltage-sensitive channels. This idea has primarily been considered with respect to aquatic animals who reside within a conductive medium (i.e., the ocean) and possess electroreceptors that are sensitive to electric stimuli as small as 5 nVcm^−1^ [43–45]. However, it is conceivable that electromagnetic induction may also occur within the endolymph of the semicircular canals of birds, with hair cells on the periphery of the cupula functioning as electroreceptors [46] (Fig 2C). If a pigeon were to rotate its head around an axis in the plane of a semicircular canal, there will be no displacement of the endolymph but electromagnetic induction could occur. Conversely, rotation around an axis perpendicular to the plane of the semicircular canal would induce fluid flow and provide information with respect to angular momentum, but would not induce electromagnetic induction [46]. In this way, inertial and electromagnetic input could be distinguished from one another. It is conceivable that the sensitivity of such a magnetoreceptor could be tuned by manipulating the concentration of cations within the endolymph, thereby altering its conductivity. We have recently discovered an iron-rich organelle, the "cuticulosome," which resides apically in avian hair cells and is composed primarily of ferritin nanoparticles [47]. Intriguingly, this structure is associated structurally and molecularly with vesicular trafficking, prompting us to propose that it may regulate the concentration of iron within inner ear compartments.

Although magnetosensation by electromagnetic induction was proposed as early as 1882 by the Frenchman Camille Viguier, there is limited behavioral, anatomical, physiological, or experimental data that directly supports it [48]. Perhaps of most relevance is the work of Dickman and colleagues who have reported that cells within the vestibular nuclei of pigeons are responsive to magnetic stimuli of a particular inclination, polarity, and intensity [49,50]. Given these data and the astonishing electrosensitivity of multiple different species on the planet, in our opinion, electromagnetic induction is an idea that deserves a closer look [51,52] (Box 2).

**Box 2. Why is research on magnetoreception so challenging?**

It is astonishing that 50 years have passed since the seminal behavioral experiments of Wiltschko and colleagues, and still our mechanistic understanding of this sense remains rudimentary. Why is this the case? Firstly, it has to be acknowledged that in contrast to many other fields in sensory biology, experimental approaches in magnetoreception cannot be based on instinctive concepts. Humans lack (at the very least) an active perception of the Earth’s magnetic field. This complicates the design of experiments that aim to unravel specific aspects of the sensory modality, and compromises an experimenter's ability to detect obvious artifacts. Secondly, electrophysiological methods, which are widely used in sensory neurobiology, have limited utility with respect to the magnetic sense. The application of magnetic stimuli risks electromagnetic induction in a recording electrode, which makes the interpretation of electrophysiological data challenging. Thirdly, magnetic fields can freely penetrate biological tissue, and therefore magnetoreceptive systems could be located in any tissue in an animal’s body, literally from head to toe [18]For those investigators exploring the magnetite-based hypothesis, this fact makes finding 20 nm-sized crystals a very challenging task [25]. Fourthly, the field has not focused on a single "model" system that can be genetically modified with ease, permitting investigators to interrogate whether a particular molecule or cell type is involved in the magnetic sense. This reflects the fact that those animals that are best at detecting magnetic fields are phylogenetically diverse, migratory, and often difficult to maintain in a laboratory setting. Fifthly, whether they rely on conditional or instinctive readouts, the establishment of behavioral paradigms that assess magnetoreception are notoriously difficult. Anything from nT strength RF interference to the chosen perfume of experimenters can render an assay useless [35,53]. Sixthly, there has been a major issue with independent replication in the field. This reflects the difficulty of the problem but also the importance of conducting all experiments in a controlled blinded fashion that are interpreted critically. Finally, it cannot be assumed that magnetoreception relies on a single receptor, or indeed a single mechanism. At conferences, Cry often faces magnetite on the scientific battlefield, but this conflict is illusionary. Selective pressure in diverse environments, from the oceans to the air, may have facilitated the evolution of a multiplicity of magnetoreceptors. This mystery might therefore have more than one solution.

### How to solve the mystery

Let’s imagine a laboratory situation in which a team of researchers aims to understand how vertebrates detect and use magnetic information. For such an endeavor to be successful, we think 2 things are imperative: (1) an interdisciplinary approach, and (2) a model system with a reliable magnetoreceptive assay that is amenable to genetic modification. If ever a scientific problem demanded the efforts of multiple disciplines, it is magnetoreception. It requires a knowledge of the physical sciences, increasingly at a quantal level, to understand the nature of the stimulus and appreciate how it can interact with biological matter. Expertise in the behavioral sciences is required to establish informative assays that exclude other sensory stimuli and enable investigators to ascertain what component of the magnetic field is influential. A detailed characterization of the neuroanatomical circuits is imperative if we are to understand where magnetic information is processed in the central nervous system. This will need to be coupled to physiological methods, such as calcium imaging, if we are to appreciate how this information is encoded and subsequently integrated with other sensory cues that are vital for navigation. The identification of the primary sensory cells, which has proved to be particularly difficult, will need a broad range of techniques including elemental analysis, electron microscopy, immunohistochemistry, live-cell imaging, and ablation studies.

Whether the underlying mechanism relies on magnetite, light, or electromagnetic induction, it is likely that the problem will then coalesce around a particular intracellular signaling pathway or molecule. At this point, the critical question will be whether a particular protein is necessary and/or sufficient for magnetoreception. To establish an iron-clad causal relationship between a molecule and a behavioral output, genetic loss (or gain) of function studies will be necessary. With the advent of the Clustered Regularly Interspaced Short Palindromic Repeats-Cas9 (CRISPR-Cas9) genome editing system, this is now a feasible option in almost any species that can be bred in captivity [54]. Although the generation of germline genetic perturbations may still be time consuming due to the reproductive cycle of a particular species, the creation of somatic genetic perturbations by in ovo or in utero electroporation is realistic in the short term [55]. Moreover, such an approach will enable tissue-specific genetic ablation that will be important if a candidate molecule (e.g., Cry 4) is ubiquitously expressed. The CRISPR-Cas9 editing system is elegantly complemented by astonishing advancements in next generation sequencing, such as the PacBio system, that enable fast, cheap, and high-quality DNA sequencing in any species. These tools, which are rapidly decreasing in cost, promise to open new experimental vistas in non-model systems. The challenge is to couple these revolutionary technologies to an informative readout, whether it be behavioral or physiological. With such an approach we are confident that this mystery, which has steadfastly refused to reveal its secrets, will succumb.

## Abbreviations

CRISPR-Cas9: Clustered Regularly Interspaced Short Palindromic Repeats-Cas9
Cry: cryptochrome
EFTEM: energy filtered transmission electron microscopy
FAD: flavin adenine dinucleotide
nT: nano-Tesla
RF: radio frequency

## References

[1] Needham J (1962) Science and Civilization in China: Cambridge University Press.

[2] Merrill RT, McElhinny M. W. (1983) The Earth's magnetic field: Its history, origin and planetary perspective. International geophysics series 32.

[3] Egevang C, Stenhouse IJ, Phillips RA, Petersen A, Fox JW, et al (2010) Tracking of Arctic terns Sterna paradisaea reveals longest animal migration. Proc Natl Acad Sci U S A 107: 2078–2081. doi: 10.1073/pnas.0909493107 20080662 10.1073/pnas.0909493107 PMC2836663

[4] Merkel FWW W. (1965) Magnetismus und richtungsfinden zugunruhiger rotkehlchen (Erithacus rubecula). Vogelwarte 23: 71–77.

[5] Blakemore R (1975) Magnetotactic bacteria. Science 190: 377–379. 170679 10.1126/science.170679

[6] Phillips JB (1986) Two magnetoreception pathways in a migratory salamander. Science 233: 765–767. 3738508 10.1126/science.3738508

[7] Lohmann KJ (1991) Magnetic orientation by hatchling loggerhead sea turtles (Caretta caretta). J Exp Biol 155: 37–49. 2016575 10.1242/jeb.155.1.37

[8] Nemec P, Altmann J, Marhold S, Burda H, Oelschlager HH (2001) Neuroanatomy of magnetoreception: the superior colliculus involved in magnetic orientation in a mammal. Science 294: 366–368. doi: 10.1126/science.1063351 11598299 10.1126/science.1063351

[9] Diebel CE, Proksch R, Green CR, Neilson P, Walker MM (2000) Magnetite defines a vertebrate magnetoreceptor. Nature 406: 299–302. doi: 10.1038/35018561 10917530 10.1038/35018561

[10] Bazalova O, Kvicalova M, Valkova T, Slaby P, Bartos P, et al (2016) Cryptochrome 2 mediates directional magnetoreception in cockroaches. Proc Natl Acad Sci U S A 113: 1660–1665. doi: 10.1073/pnas.1518622113 26811445 10.1073/pnas.1518622113 PMC4760799

[11] Coey JMD (2010) Magnetism and Magnetic Materials: Cambridge University Press.

[12] Block SM (1992) Biophysical principles of sensory transduction. Soc Gen Physiol Ser 47: 1–17. 1369757

[13] Yoshioka T, Sakakibara M (2013) Physical aspects of sensory transduction on seeing, hearing and smelling. Biophysics (Nagoya-shi) 9: 183–191. 27493557 10.2142/biophysics.9.183 PMC4629681

[14] Syeda R, Florendo MN, Cox CD, Kefauver JM, Santos JS, et al (2016) Piezo1 Channels Are Inherently Mechanosensitive. Cell Rep 17: 1739–1746. doi: 10.1016/j.celrep.2016.10.033 27829145 10.1016/j.celrep.2016.10.033 PMC5129625

[15] Ranade SS, Syeda R, Patapoutian A (2015) Mechanically Activated Ion Channels. Neuron 87: 1162–1179. doi: 10.1016/j.neuron.2015.08.032 26402601 10.1016/j.neuron.2015.08.032 PMC4582600

[16] Winklhofer M, Kirschvink JL (2010) A quantitative assessment of torque-transducer models for magnetoreception. J R Soc Interface 7 Suppl 2: S273–289. 10.1098/rsif.2009.0435.focus PMC2843997 20086054

[17] Uebe R, Schuler D (2016) Magnetosome biogenesis in magnetotactic bacteria. Nat Rev Microbiol 14: 621–637. doi: 10.1038/nrmicro.2016.99 27620945 10.1038/nrmicro.2016.99

[18] Johnsen S, Lohmann KJ (2005) The physics and neurobiology of magnetoreception. Nat Rev Neurosci 6: 703–712. doi: 10.1038/nrn1745 16100517 10.1038/nrn1745

[19] Walker MM (2008) A model for encoding of magnetic field intensity by magnetite-based magnetoreceptor cells. J Theor Biol 250: 85–91. doi: 10.1016/j.jtbi.2007.09.030 18028964 10.1016/j.jtbi.2007.09.030

[20] Treiber CD, Salzer MC, Riegler J, Edelman N, Sugar C, et al (2012) Clusters of iron-rich cells in the upper beak of pigeons are macrophages not magnetosensitive neurons. Nature. 10.1038/nature11046 22495303

[21] Fleissner G, Holtkamp-Rotzler E, Hanzlik M, Winklhofer M, Petersen N, et al (2003) Ultrastructural analysis of a putative magnetoreceptor in the beak of homing pigeons. J Comp Neurol 458: 350–360. doi: 10.1002/cne.10579 12619070 10.1002/cne.10579

[22] Eder SH, Cadiou H, Muhamad A, McNaughton PA, Kirschvink JL, et al (2012) Magnetic characterization of isolated candidate vertebrate magnetoreceptor cells. Proc Natl Acad Sci U S A 109: 12022–12027. doi: 10.1073/pnas.1205653109 22778440 10.1073/pnas.1205653109 PMC3409731

[23] Edelman NB, Fritz T, Nimpf S, Pichler P, Lauwers M, et al (2015) No evidence for intracellular magnetite in putative vertebrate magnetoreceptors identified by magnetic screening. Proc Natl Acad Sci U S A 112: 262–267. doi: 10.1073/pnas.1407915112 25535350 10.1073/pnas.1407915112 PMC4291630

[24] Kobayashi AK, Kirschvink JL, Nesson MH (1995) Ferromagnetism and EMFs. Nature 374: 123 doi: 10.1038/374123a0 7877680 10.1038/374123a0

[25] Shaw J, Boyd A, House M, Woodward R, Mathes F, et al (2015) Magnetic particle-mediated magnetoreception. J R Soc Interface 12: 0499 doi: 10.1098/rsif.2015.0499 26333810 10.1098/rsif.2015.0499 PMC4614459

[26] Jones SR, Wilson TD, Brown ME, Rahn-Lee L, Yu Y, et al (2015) Genetic and biochemical investigations of the role of MamP in redox control of iron biomineralization in Magnetospirillum magneticum. Proc Natl Acad Sci U S A 112: 3904–3909. doi: 10.1073/pnas.1417614112 25775527 10.1073/pnas.1417614112 PMC4386411

[27] Taoka A, Eguchi Y, Mise S, Oestreicher Z, Uno F, et al (2014) A magnetosome-associated cytochrome MamP is critical for magnetite crystal growth during the exponential growth phase. FEMS Microbiol Lett 358: 21–29. doi: 10.1111/1574-6968.12541 25048532 10.1111/1574-6968.12541

[28] Palczewski K (2012) Chemistry and biology of vision. J Biol Chem 287: 1612–1619. doi: 10.1074/jbc.R111.301150 22074921 10.1074/jbc.R111.301150 PMC3265841

[29] Wiltschko W, Wiltschko R (2001) Light-dependent magnetoreception in birds: the behaviour of European robins, Erithacus rubecula, under monochromatic light of various wavelengths and intensities. J Exp Biol 204: 3295–3302. 11606603 10.1242/jeb.204.19.3295

[30] Ritz T, Adem S, Schulten K (2000) A model for photoreceptor-based magnetoreception in birds. Biophys J 78: 707–718. doi: 10.1016/S0006-3495(00)76629-X 10653784 10.1016/S0006-3495(00)76629-X PMC1300674

[31] Hore PJ, Mouritsen H (2016) The Radical-Pair Mechanism of Magnetoreception. Annu Rev Biophys 45: 299–344. doi: 10.1146/annurev-biophys-032116-094545 27216936 10.1146/annurev-biophys-032116-094545

[32] Schulten K SC, Weller A (1978) A biomagnetic sensory mechanism based on magnetic field modulated coherent electron spin motion. Z Phys Chem 111: 1–5.

[33] Timmel CR, Henbest KB (2004) A study of spin chemistry in weak magnetic fields. Philos Transact A Math Phys Eng Sci 362: 2573–2589. 10.1098/rsta.2004.1459 15539359

[34] Henbest KB, Maeda K, Hore PJ, Joshi M, Bacher A, et al (2008) Magnetic-field effect on the photoactivation reaction of Escherichia coli DNA photolyase. Proc Natl Acad Sci U S A 105: 14395–14399. doi: 10.1073/pnas.0803620105 18799743 10.1073/pnas.0803620105 PMC2567148

[35] Engels S, Schneider NL, Lefeldt N, Hein CM, Zapka M, et al (2014) Anthropogenic electromagnetic noise disrupts magnetic compass orientation in a migratory bird. Nature 509: 353–356. doi: 10.1038/nature13290 24805233 10.1038/nature13290

[36] Mouritsen H, Ritz T (2005) Magnetoreception and its use in bird navigation. Curr Opin Neurobiol 15: 406–414. doi: 10.1016/j.conb.2005.06.003 16006116 10.1016/j.conb.2005.06.003

[37] Fedele G, Edwards MD, Bhutani S, Hares JM, Murbach M, et al (2014) Genetic analysis of circadian responses to low frequency electromagnetic fields in Drosophila melanogaster. PLoS Genet 10: e1004804 doi: 10.1371/journal.pgen.1004804 25473952 10.1371/journal.pgen.1004804 PMC4256086

[38] Haque R, Chaurasia SS, Wessel JH 3rd, Iuvone PM (2002) Dual regulation of cryptochrome 1 mRNA expression in chicken retina by light and circadian oscillators. Neuroreport 13: 2247–2251. doi: 10.1097/01.wnr.0000044218.09266.c9 12488805 10.1097/00001756-200212030-00016

[39] Vitaterna MH, Selby CP, Todo T, Niwa H, Thompson C, et al (1999) Differential regulation of mammalian period genes and circadian rhythmicity by cryptochromes 1 and 2. Proc Natl Acad Sci U S A 96: 12114–12119. 10518585 10.1073/pnas.96.21.12114 PMC18421

[40] Fritzsch B, Straka H (2014) Evolution of vertebrate mechanosensory hair cells and inner ears: toward identifying stimuli that select mutation driven altered morphologies. J Comp Physiol A Neuroethol Sens Neural Behav Physiol 200: 5–18. doi: 10.1007/s00359-013-0865-z 24281353 10.1007/s00359-013-0865-z PMC3918741

[41] Hudspeth AJ, Tanaka K (1998) Sensory systems. Curr Opin Neurobiol 8: 443–446. 9751666 10.1016/s0959-4388(98)80029-9

[42] Faraday M (1832) Experimental Researches in Electricity. Philosophical Transactions of the Royal Society of London 122: 125–162.

[43] Kalmijn AJ (1971) The electric sense of sharks and rays. J Exp Biol 55: 371–383. 5114029 10.1242/jeb.55.2.371

[44] Kalmijn AJ (1982) Electric and magnetic field detection in elasmobranch fishes. Science 218: 916–918. 7134985 10.1126/science.7134985

[45] Meyer CG, Holland KN, Papastamatiou YP (2005) Sharks can detect changes in the geomagnetic field. J R Soc Interface 2: 129–130. doi: 10.1098/rsif.2004.0021 16849172 10.1098/rsif.2004.0021 PMC1578252

[46] Jungerman RL, Rosenblum B (1980) Magnetic induction for the sensing of magnetic fields by animals—an analysis. J Theor Biol 87: 25–32. 7206749 10.1016/0022-5193(80)90217-9

[47] Lauwers M, Pichler P, Edelman NB, Resch GP, Ushakova L, et al (2013) An iron-rich organelle in the cuticular plate of avian hair cells. Curr Biol 23: 924–929. doi: 10.1016/j.cub.2013.04.025 23623555 10.1016/j.cub.2013.04.025

[48] Viguier C (1882) Le Sens De L'Orientation et ses Organes. Revue Philosophique: 1–36.

[49] Wu LQ, Dickman JD (2012) Neural correlates of a magnetic sense. Science 336: 1054–1057. doi: 10.1126/science.1216567 22539554 10.1126/science.1216567

[50] Wu LQ, Dickman JD (2011) Magnetoreception in an avian brain in part mediated by inner ear lagena. Curr Biol 21: 418–423. doi: 10.1016/j.cub.2011.01.058 21353559 10.1016/j.cub.2011.01.058 PMC3062271

[51] Bellono NW, Leitch DB, Julius D (2017) Molecular basis of ancestral vertebrate electroreception. Nature 543: 391–396. doi: 10.1038/nature21401 28264196 10.1038/nature21401 PMC5354974

[52] Clarke D, Whitney H, Sutton G, Robert D (2013) Detection and learning of floral electric fields by bumblebees. Science 340: 66–69. doi: 10.1126/science.1230883 23429701 10.1126/science.1230883

[53] Pinzon-Rodriguez A, Muheim R (2017) Zebra finches have a light-dependent magnetic compass similar to migratory birds. J Exp Biol 220: 1202–1209. doi: 10.1242/jeb.148098 28356366 10.1242/jeb.148098

[54] Reardon S (2016) Welcome to the CRISPR zoo. Nature 531: 160–163. doi: 10.1038/531160a 26961640 10.1038/531160a

[55] Veron N, Qu Z, Kipen PA, Hirst CE, Marcelle C (2015) CRISPR mediated somatic cell genome engineering in the chicken. Dev Biol 407: 68–74. doi: 10.1016/j.ydbio.2015.08.007 26277216 10.1016/j.ydbio.2015.08.007

